# Development of a novel prediction method of *cis*-elements to hypothesize collaborative functions of *cis*-element pairs in iron-deficient rice

**DOI:** 10.1186/1939-8433-6-22

**Published:** 2013-09-22

**Authors:** Yusuke Kakei, Yuko Ogo, Reiko N Itai, Takanori Kobayashi, Takashi Yamakawa, Hiromi Nakanishi, Naoko K Nishizawa

**Affiliations:** Graduate School of Agricultural and Life Sciences, The University of Tokyo, 1-1-1 Yayoi, 113-8657 Bunkyo-ku Tokyo, Japan; Research Institute for Bioresources and Biotechnology, Ishikawa Prefectural University, 1-308 Suematsu, 921-8836 Nonoichi-machi, Ishikawa Japan; Plant Biotechnology Division, Yokohama City University, Kihara Institute for Biological Research Maiokacho, 641-12, Totsuka, Yokohama, Kanagawa 244-0813 Japan; Functional Transgenic Crops Research Unit, Genetically Modified Organism Research Center National Institute of Agrobiological Sciences, Kannondai 2-1-2, 305-8602 Tsukuba, Ibaraki Japan; Research Institute for Bioresources and Biotechnology, Ishikawa Prefectural University, 1-308 Suematsu, 921-8836 Nonoichi-machi, Ishikawa Japan

**Keywords:** *Cis*-element, Iron deficiency, Transcription

## Abstract

**Background:**

*Cis*-acting elements are essential genomic sequences that control gene expression. In higher eukaryotes, a series of *cis*-elements function cooperatively. However, further studies are required to examine the co-regulation of multiple *cis*-elements on a promoter. The aim of this study was to propose a model of *cis*-element networks that cooperatively regulate gene expression in rice under iron (Fe) deficiency.

**Results:**

We developed a novel clustering-free method, microarray-associated motif analyzer (MAMA), to predict novel *cis*-acting elements based on weighted sequence similarities and gene expression profiles in microarray analyses. Simulation of gene expression was performed using a support vector machine and based on the presence of predicted motifs and motif pairs. The accuracy of simulated gene expression was used to evaluate the quality of prediction and to optimize the parameters used in this method. Based on sequences of *Oryza sativa* genes upregulated by Fe deficiency, MAMA returned experimentally identified *cis*-elements responsible for Fe deficiency in *O. sativa*. When this method was applied to *O. sativa* subjected to zinc deficiency and *Arabidopsis thaliana* subjected to salt stress, several novel candidate *cis*-acting elements that overlap with known *cis*-acting elements, such as ZDRE, ABRE, and DRE, were identified. After optimization, MAMA accurately simulated more than 87% of gene expression. Predicted motifs strongly co-localized in the upstream regions of regulated genes and sequences around transcription start sites. Furthermore, in many cases, the separation (in bp) between co-localized motifs was conserved, suggesting that predicted motifs and the separation between them were important in the co-regulation of gene expression.

**Conclusions:**

Our results are suggestive of a typical sequence model for Fe deficiency-responsive promoters and some strong candidate *cis*-elements that function cooperatively with known *cis*-elements.

**Electronic supplementary material:**

The online version of this article (doi:10.1186/1939-8433-6-22) contains supplementary material, which is available to authorized users.

## Background

Gene expression is regulated by various factors, including transcription factors (TFs), *cis*-acting elements, cofactors, and chromatin structure, and by processes such as methylation and acetylation. Many *cis*-acting elements essential for the regulation of gene expression have been identified, mostly upstream of transcribed sequences. Many reports have described transcription factors regulating gene expression by functionally coordinating with *cis*-elements (Raff and Kaufman 1991; Wilkins 1991; Gerhart and Kirschner 1997; Carroll et al. 2001) and binding to specific sites (Levine and Tjian 2003).

For more than 10 years, during which time a variety of genomes have been fully sequenced, much effort has been devoted to the development of *in silico* methods for predicting novel *cis*-acting sequences or motifs in prokaryotes and eukaryotes. These methods are categorized into two general groups (Hudson and Quail 2003; van Hijum et al. 2009): alignment (probabilistic) methods, such as MEME (Bailey and Elkan 1994), DMBP (Huang et al. 2005), AlignACE (Hughes et al. 2000), and Motif Sampler (Thijs et al. 2001), and enumerative methods (Hudson and Quail 2003; van Hijum et al. 2009). In prokaryotes, noncoding regions are typically short, and *cis*-elements are highly accumulated (Gama-Castro et al. 2008). Thus, existing methods can often correctly predict *cis*-elements in prokaryotes. In contrast, in eukaryotic genomes (especially higher eukaryotes such as humans and rice) the noncoding regions are much longer, which is believed to be one main reason as to why prediction in higher eukaryotes is more difficult. Additionally, many *cis*-elements co-localize in the long upstream sequences and cooperate in the regulation of gene transcription (Carrera and Treisman 2008). Vandenbon et al. (2012) reported that some *cis*-elements co-localize significantly in the fly genome; of these identified, they experimentally validated the co-regulation of a pair of binding sites within NF-κB and C/EBP. Therefore, predicting a series of *cis*-elements that function cooperatively has become increasingly important to understand transcriptional regulation in higher eukaryotes.

Alignment methods are designed to find commonalities in a group of upstream sequences, primarily by aligning similar sequences and creating a probabilistic model, such as a position–weight matrix. Alignment methods are often impaired by “false predictions” caused by the ubiquitously present short sequences throughout the genome. For example, A/T-repeats (e.g., AAAAAA) are often predicted. Such A/T-repeat sequences are known to be common in intergenic regions, although they are not known to be included in transcription. In enumerative methods, numbers of all the small sequences in a group of upstream sequences are counted and compared with those in a background group. They usually do not evaluate sequence similarity, although many *cis*-acting sequences are reportedly quite fuzzy (Collado-Vides et al. 1991). Clustering (i.e., grouping of similarly expressed genes) plays a key role in the prediction of *cis*-motif elements in both alignment and enumerative methods. However, clustering genes is difficult. For example, clustered genes do not always share the same *cis*-elements, and selection of the best thresholds in clustering is a difficult issue (Kundaje et al. 2007). Some clustering-free methods are available: REDUCE (Bussemaker et al. 2007) and a method by S.-Y. Kim and Kim (2006) use genome-wide gene expression as input without clustering. However, REDUCE is not applicable to plants, and the method by Kim and Kim (2006) is not designed to predict novel *cis*-motif elements.

The regulatory mechanisms of iron (Fe) deficiency-inducible genes were explored using molecular biological and plant physiological approaches in rice. We reported that Fe deficiency-responsive element 1 (IDE1: ATCAAGCATGCTTCTTGC) and IDE2 (TTGAACGGCAAGTTTCACGCTGTCACT) were critical *cis*-elements for several genes upregulated by Fe deficiency (Kobayashi et al. 2003). We also identified the transcription factors that associate with IDE1 and IDE2 (IDEF1, IDEF2; Kobayashi et al. 2007; Ogo et al. 2008). Furthermore, one of the Fe deficiency-inducible transcription factors, OsIRO2, was analyzed, and its binding sequence was investigated (Ogo et al. 2007). The TF-binding sequences (TFBSs) of these TFs are found in only 20–60% of genes regulated under Fe deficiency (Kobayashi et al. 2009), suggesting that novel *cis*-elements remain to be discovered. IDEF1 function as a master regulator in rice under iron deficiency. Therefore to find the other *cis*-elements function cooperatively with IDEF1-binding sequence is especially important.

To identify novel *cis*-acting motifs in Fe deficiency-induced genes in rice, we applied existing motif prediction methods, that is, MEME (Bailey and Elkan 1994), Motif Sampler (Thijs et al. 2001), and SIFT (Hudson and Quail 2003), to some different number of genes upregulated by Fe deficiency (results with the top 50 genes are shown in Additional file 1 online). However, transcription factor-binding sequences (i.e., IDEF1, IDEF2, and OsIRO2) were predicted after dozens of sequences were predicted as “more likely to be *cis*-elements” (according to their Higher Highest II, lower E-value, and *P*-values). These methods are designed to identify commonly shared *cis*-motifs from clustered genes. Under iron-deficient condition, OsIRO2 is regulated by IDEF1 (Kobayashi et al. 2009) and OsIRO2 regulates the expression of some other TFs (Ogo et al. 2007). Therefore, it was expected that this regulatory cascade of TFs makes it difficult to make a cluster of genes sharing common *cis*-elements. Iron-deficiency regulated genes may not have highly common *cis*-elements but they should have one of the binding sequences of IDEF1, IDEF2, OsIRO2 and other TFs regulated by OsIRO2. This failure motivated us to develop a novel prediction method able to extract functional *cis*-acting elements without clustering.

To effectively predict *cis*-motifs in eukaryotes, we developed a novel *in silico* method, which we named microarray-associated motif analyzer (MAMA). This method generates an *ab initio* prediction of *cis*-elements, which are independent from the predictions by existing methods. We attempted to evaluate the frequency of sequences that specifically exist in upregulated genes, the degree of mismatch and identity, and degree of gene expression without clustering using a MAMA score (Additional file 2). MAMA was applied to the microarray data in rice subjected to Fe deficiency, and the accumulation of motif pairs was also evaluated using this method. We found that the distribution and co-localization of predicted motifs are often conserved in the promoter region of treatment-regulated genes. MAMA was also applied to other microarray data of rice subjected to Zn-deficiency treatment and *Arabidopsis thaliana* subjected to NaCl.

## Results and discussion

### Development of the MAMA method and its application to O. sativa

**Figure 1:**
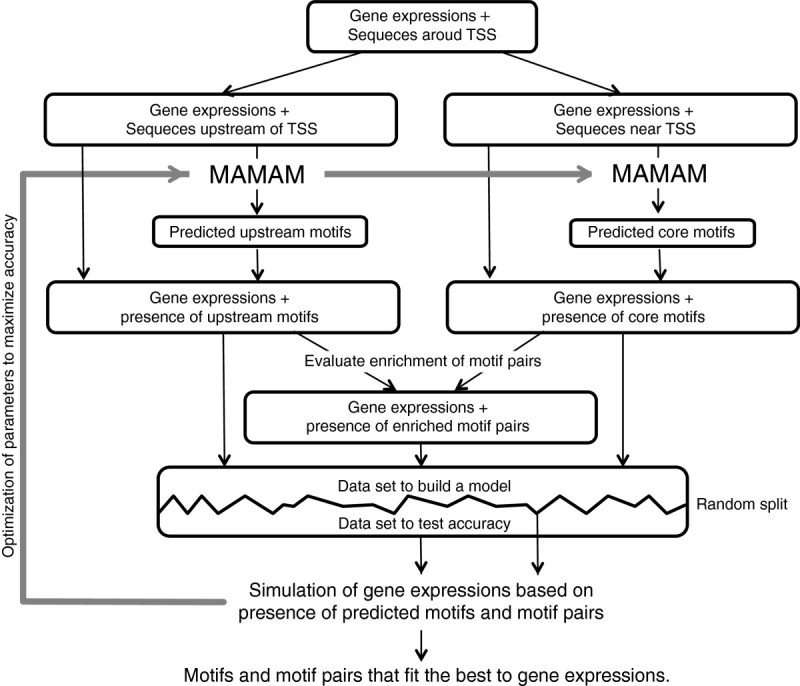
**Flowchart summary of the MAMA method.** MAMAM is the main software of MAMA.

The main flowchart of this MAMA method is shown in Figure 1. This method predicts *cis*-acting motifs based on a MAMA score calculated from similarities in sequence and gene expression profiles in microarray analyses (Methods, Additional file 2). Since most TFBSs reported are less than 8 bp in length, this method initially lists every 8-bp sequence upstream of regulated genes as candidate sequences. Subsequently, all candidate sequences are compared with each upstream sequence of all genes to identify the most similar sequence in each upstream sequence. These similarities are calculated as *h_scores*. The enrichment of similar sequences in treatment-regulated genes was evaluated as MAMA scores of the candidate sequences. If part of the candidate sequence is frequently observed in highly regulated genes, the MAMA score will increase. Overrepresented candidate sequences that attained a high score and are similar to each other are aligned and grouped into motifs. Subsequently, the presence of predicted motifs is investigated on sequences of all genes, and their presence and absence is used for gene expression simulation, in which all gene expression and sequence data are randomly divided into two. One is used for the construction of a model used to simulate transcription regulation based on the presence of motifs and motif pairs, while the other is used to evaluate the accuracy of the model. The accuracy of the simulation model was used to optimize the parameters used in MAMA (Figure 1, Methods). Initially, the 500 bp immediately upstream of transcription start sites (TSSs) was used for analyses. TFBS, IDEF1, and similar sequences are commonly found 500 bp immediately upstream of the TSS (Kobayashi et al. 2009).

**Figure 2:**
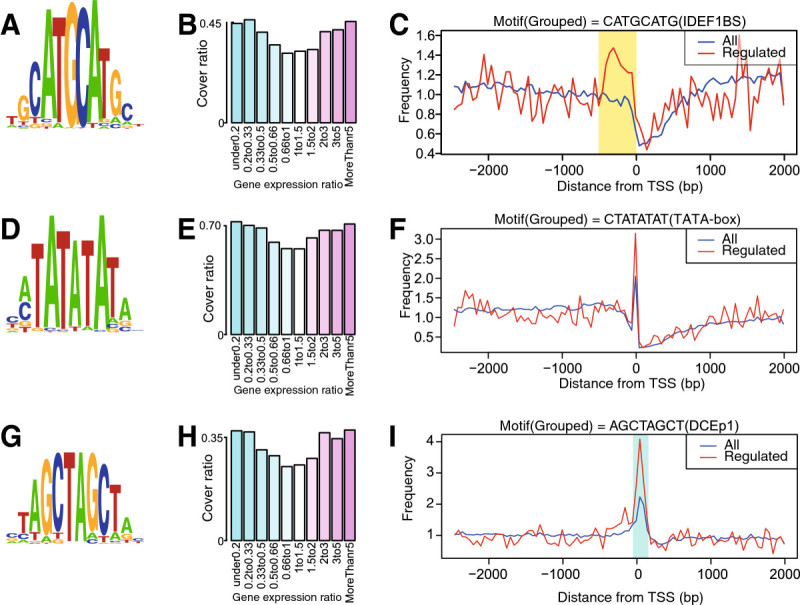
**Characterization of motifs predicted using data from rice roots subjected to iron (Fe) deficiency. A**, Logo represents aligned sequences included in the CATGCATG motif (IDEF1BS motif). Heights of A/C/G/T in the logo represent the frequency of bases at that position. **B**, Cover ratio (*CR*) of the IDEF1BS motif in the 500-bp upstream regions. **C**, Distribution of the IDEF1BS motif. Numbers of IDEF1BS motifs were counted at a region from 3,000 bp upstream to 2,000 bp downstream of the TSS. The blue line represents the frequency of IDEF1BS in all genes. The number of IDEF1BS motifs in a 50-bp window in genes upregulated by Fe deficiency (red) or all genes (blue) divided by the number of upregulated (*N*(UP) = 895) or all genes (*N* = 31,348); next, the frequency was normalized by the average frequency of all genes from 3,000 bp upstream to 2,000 bp downstream of the TSS. **D**, Logo of the CTATATAT motif (TATA-box motif). **E**, Cover ratio of the TATA-box motif. **F**, Distribution of the TATA-box motif. **G**, Logo of the AGCTAGCT motif (DCEp1 motif). **H**, Cover ratio of the DCEp1 motif in the 500-bp upstream regions. **I**, Distribution of the DCEp1 motif.

We applied MAMA to microarray data of rice roots under Fe-deficient and -sufficient conditions to predict *cis*-elements responsive to Fe deficiency. The top 30 high-scoring motifs are shown in Table 1. The CATGCATG motif, which contains an IDEF1-binding sequence (CATGC), was predicted with the fourth highest MAMA score (Table 1). We named this motif IDEF1-binding sequence-containing motif (IDEF1BS). Grouped sequences included in IDEF1BS were aligned and converted into a logo (Figure 2A). IDEF1BS was included in 45% of upstream sequences of genes whose expressions were increased over fivefold by Fe deficiency. This consists with a previous report that the binding sequence of IDEF1 exists about 20-60% of iron-regulated genes (Kobayashi et al. 2009). In contrast, it was included in only 35% of upstream sequences of genes whose expression did not change (>0.66–1.5-fold; Figure 2B). Furthermore, IDEF1BS was found most frequently 500 bp upstream from the TSSs of genes that were induced more than twofold by Fe deficiency, whereas IDEF1BS was not common within 500 bp upstream of the TSSs of all genes (Figure 2C). The CGCCACGT motif, which contains the OsIRO2-binding sequence (CACGTGG) and was named OsIRO2-binding sequence-containing motif (OsIRO2BS), attained the sixth highest MAMA score. The CAAGAATC motif, which contains the IDEF2-binding sequence (CA[A/C]G[T/C][T/C/A][T/C/A]) and was named IDEF2-binding sequence-containing motif (IDEF2BS), had the eleventh highest MAMA score (Table 1, Additional file 3 online). OsIRO2BS and IDEF2BS were also specifically overrepresented in genes upregulated by Fe deficiency, and they were observed frequently within 500 bp upstream of TSSs (Additional file 4 online).

**Table 1:** **Motifs predicted by MAMA using microarray data from iron-deficient and -sufficient rice roots**

Motif name	Score	***P***-value*	Annotations
ACGTACGT	1.881	3.03E-34	**FAM1 motif**
AGCTAGCT	1.880	2.73E-51	**DCEp1 motif**
CTATATAT	1.872	<1.0E-300	**TATA-box motif**
CATGCATG	1.867	<1.0E-300	**IDEF1BS motif**
CTAGCAGA	1.865	8.45E-13	
CGCCACGT	1.862	6.57E-23	**OsIRO2BS motif**
AGTCAACT	1.860	7.54E-19	
TGATCAAC	1.854	1.20E-13	
ACTACGTA	1.853	1.21E-09	
GCATGCTG	1.850	2.80E-10	Motif containing IDEF1 binding sequence
CAAGAATC	1.848	1.63E-09	**IDEF2BS motif**
CGCCTATA	1.845	1.34E-09	**BRE** ^**U**^ **-TATA motif 1**
TAGCTGCA	1.845	2.10E-06	
TGGCGACA	1.843	1.88E-17	
GCGCGCTA	1.843	4.18E-12	
TAGCAAGT	1.842	8.53E-14	
ACTGTAGC	1.838	2.22E-07	
GTAGTACG	1.837	1.08E-04	
ATGGCCAT	1.837	1.43E-13	
CCTGAAGA	1.837	4.04E-04	
GAACGTGT	1.836	1.06E-07	
CATCAGCA	1.835	8.28E-12	
TCGACGTG	1.834	2.34E-04	
ATTAAGCG	1.833	3.43E-06	
CTGGCACT	1.833	3.33E-04	
TACTAGTA	1.831	3.89E-05	
GCATATGC	1.83	3.16E-05	
GTGACGTC	1.829	2.36E-03	
AATACTCT	1.828	1.18E-07	

* *P*-values were calculated using a binominal test. Using 500 bp of upstream sequence from the TSS, the number of motifs in regulated genes and the number of every 8-bp sequence in regulated genes were compared from the number of motifs in all genes and the number of every 8-bp sequence in all genes. This *P*-value was not used to predict *cis*-acting motifs. These numbers used to calculate *P*-values and annotations using PLACE and TRANSFAC are shown in Additional file 3.

The CTATATAT motif recorded the third highest MAMA score (Table 1, Figure 2D) and was named the TATA-box motif. The TATA-box motif existed most frequently within 50 bp upstream of TSSs of genes that were induced more than twofold by Fe deficiency (Figure 2E, F). The TATA-box motif was also common upstream of genes whose expression was decreased less than 0.5-fold (Figure 2E). Several novel motifs that have not been reported to be related to Fe-deficiency responses were found to have high MAMA scores (Table 1). In particular, the ACGTACGT motif was predicted with the highest MAMA score (Table 1). We named this motif Fe deficiency-associated motif 1 (FAM1). FAM1 was frequently found within 500 bp upstream of TSSs of genes upregulated by Fe deficiency (Additional file 4 I online).

### Motifs immediately downstream of TSSs

Among the predicted motifs (Table 1), AGCTAGCT was strongly conserved immediately downstream of TSSs (Figure 2G–I). To accurately predict *cis*-motifs, MAMA was also applied to this region from 50 bp upstream to 150 bp downstream of the TSSs to identify common motifs. The TATA-box motif recorded the highest MAMA score, but it was overrepresented only upstream of TSSs (Table 2, Additional file 5 online). The AGCTAGCT motif recorded the second highest MAMA score (Table 2). We named this motif putative downstream core element 1 (DCEp1). We found that the sequences CGCC and GCC were often attached to a TATA-box sequence upstream of Fe deficiency-upregulated genes. The CGCCTATA (Table 1) and GCCTATAA (Table 2) motifs recorded the thirteenth and sixth highest MAMA scores, respectively. TFIIB-recognition elements (BREs) are known to attach to the TATA box in yeast (Deng and Roberts 2006). One of these, upstream BRE (BRE^U^; C[C/G][C/G]GCC), was similar to the CGCC and GCC attached to TATA-box sequences. We named these motifs BRE^U^-TATA motif 1 and BRE^U^-TATA motif 2, respectively (Tables 1, 2).

**Table 2:** **Motifs predicted from a region 50 bp upstream to 150 bp downstream of TSS**

Motif name	Sscore	***P***-value*	Annotations
CTATATAT	2.08	1.03E-69	**TATA-box motif**
AGCTAGCT	2.05	1.71E-86	**DCEp1 motif**
TATAAGTA	2.00	3.56E-05	
CTTAATTA	1.99	3.68E-25	
TGATCATG	1.99	2.57E-12	
GCCTATAA	1.97	9.44E-08	**BRE** ^**U**^ **-TATA motif 2**
TATACACA	1.96	3.47E-15	
TATAAAAG	1.95	1.19E-05	
TAACTAGT	1.95	4.19E-10	
GTCCTGTA	1.95	2.09E-08	
CAACTATA	1.95	2.56E-07	
CACTTAGT	1.94	6.14E-06	
ACTGAAGT	1.93	6.06E-05	
CATCAAGC	1.93	2.22E-08	
GTACTACG	1.93	1.74E-07	
ACATACCA	1.93	7.81E-09	
AGTTGCAG	1.93	1.93E-11	
GTACGTTC	1.93	7.25E-10	
GCTATAGC	1.92	3.32E-08	
CTAAGCTA	1.92	2.17E-09	

* *P*-values were calculated using a binominal test. Using 500 bp of upstream sequence from the TSS, the number of motifs in regulated genes and the number of every 8-bp sequence in regulated genes were compared from the number of motifs in all genes and the number of every 8-bp sequence in all genes. This *P*-value was not used to predict *cis*-acting motifs. These numbers used to calculate *P*-values and annotations using PLACE and TRANSFAC are shown in Additional file 5.

### Co-localization of predicted motifs in upregulated genes

**Figure 3:**
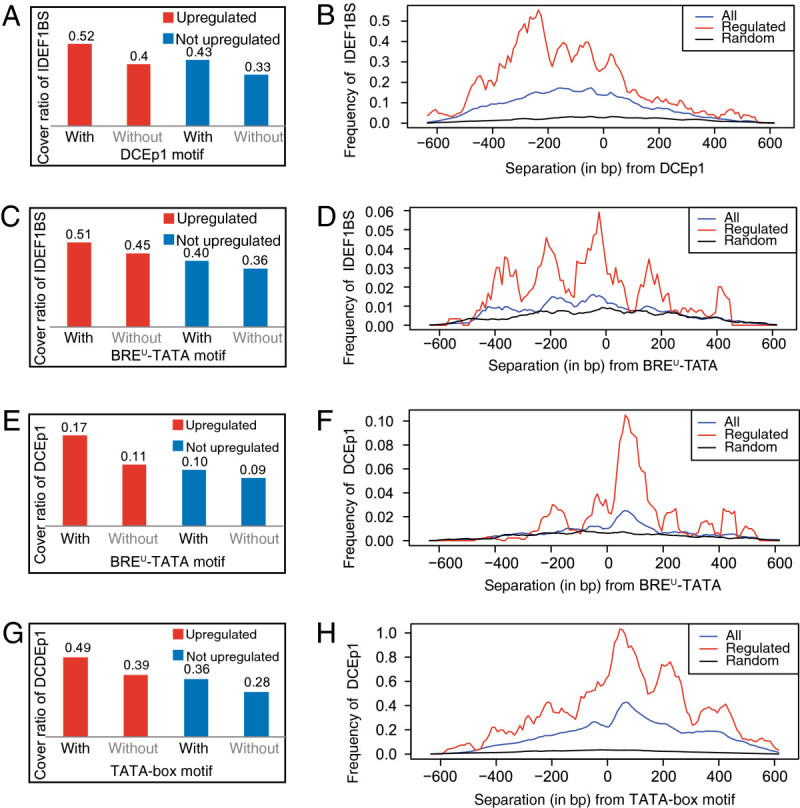
**Separations (in bp) between motifs extracted by MAMA. A**, Cover ratio of the IDEF1BS motif with (*CR*(IDEF1BS|DCEp1|UP), *CR*(IDEF1BS|DCEp1|!UP)) and without the DCEp1 motif (*CR*(IDEF1BS|!DCEp1|UP), *CR*(IDEF1BS|!DCEp1|!UP)). **B**, Separations between the IDEF1BS and DCEp1 motifs. Separations between the IDEF1BS and DCEp1 motifs were calculated, and the number of IDEF1BS motifs co-localizing with DCEp1 motifs in a region 500 bp upstream and 150 bp downstream of the TSSs was counted. Black lines, frequency of motifs in random and 650-bp sequences; blue lines, number of motifs in all genes; red lines, number of motifs in genes upregulated over twofold in response to iron deficiency. Frequency represents the number of IDEF1BS motifs in each ±25-bp window in random sequences, upregulated genes, and all genes divided by the number of random sequences (31,348), upregulated genes (895), and all genes (31,348). **C**, Cover ratio of the IDEF1BS motif with the BRE^U^-TATA motif 1. **D**, Separations between the IDEF1BS motif and BRE^U^-TATA motif 1. **E**, Cover ratio of the DCEp1 motif with the BRE^U^-TATA motif 1. **F**, Separations between the DCEp1 motif and BRE^U^-TATA motif 1. **G**, Cover ratio of the DCEp1 motif with the TATA-box motif. **H**, Separations between the DCEp1 motif and TATA-box motif.

Some combinations of MAMA-predicted motifs displayed strong co-localization in a region 500 bp upstream to 150 bp downstream of TSSs (Additional file 6) and showed unique patterns of separation (in bp). Among the rice genes upregulated by Fe deficiency, 52% of sequences containing DCEp1 motifs also contained an IDEF1BS motif, although only 43% of sequences without a DCEp1 contained an IDEF1BS (Figure 3A). In genes not upregulated by Fe deficiency, 42% and 33% of sequences with and without a DCEp1 motif, respectively, contained an IDEF1BS. Upregulated genes that contained a DCEp1 motif possessed IDEF1BS significantly more often than non-upregulated genes (χ^2^ test; *P* < 0.01). In genes upregulated by Fe deficiency with a DCEp1 motif, IDEF1BS motifs occurred at a high frequency upstream of the DCEp1 motif (Figure 3B). Moreover, they were most commonly noted at approximately ±50 bp relative to the DCEp1 motif, 150 and 250 upstream of the DCEp1 motif (Figure 3B). The BRE^U^-TATA motif 1 also significantly co-localized with the IDEF1BS (*P* < 0.01; Figure 3C) and the DCEp1 (*P* < 0.01; Figure 3E) motifs in upregulated genes. IDEF1BS motifs were specifically overrepresented at around 50, 200, and 350 bp upstream of the BRE^U^-TATA motif 1 in upregulated genes (Figure 3D). Elsewhere, DCEp1 motifs were most commonly observed approximately 80 bp downstream of BRE^U^-TATA motif 1 (Figure 3F). TATA-box motifs and DCEp1 co-localized in 52% of sequences around the TSS of genes upregulated more than twofold (Figure 3G), but in only 27% of genes whose induction was less than 1.5-fold. DCEp1 motifs were specifically overrepresented at approximately 80, 220, and 400 bp upstream of the TATA-box motif 1 in upregulated genes (Figure 3H).

### MAMA successfully returned known cis-elements from rice roots subjected to zinc deficiency

**Figure 4:**
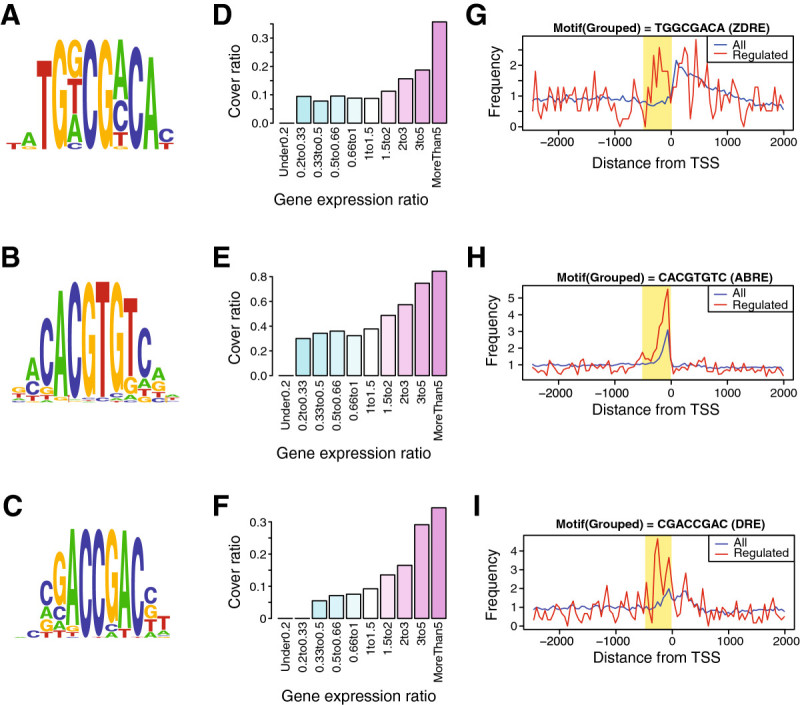
**Predicted motifs from other microarrays. A**, The TGGCGACA motif (containing a ZDRE) attained the highest MAMA score when microarray data generated from rice roots subjected to zinc deficiency were analyzed. **B**, The CACGTGTC motif (containing the ABRE consensus) attained the highest MAMA score when microarray data generated from *Arabidopsis* roots subjected to NaCl stress were analyzed. **C**, The CGACCGAC motif (which contains a DRE) attained the highest MAMA score when human microarray data generated from *Arabidopsis* roots subjected to NaCl stress were analyzed. **D**, Cover ratios of the TGGCGACA motif. **E**, Cover ratios of the CACGTGTC motif. **F**, Cover ratios of the CGACCGAC motif. **G**, Distribution of the TGGCGACA motif (upregulated genes, 223; all genes, 29,535). **H**, Distribution of the ACACGTGT motif (upregulated genes, 335; all genes, 20,767). **I**, Distribution of the CGACCGAC motif.

To investigate whether MAMA can predict *cis*-elements in other microarray data, we applied it to microarray data from rice root subjected to zinc (Zn) deficiency (Suzuki et al. 2012). The motif contained the last 8 bp of a Zn-deficiency response element (ZDRE; ATGTCGACA); a *cis*-element responsive for Zn deficiency (Assunção et al. 2010) yielded the thirteenth highest MAMA score (Figure 4A, Additional file 7 online). Motifs including ZDRE were found at particularly high frequencies within 500 bp upstream of the TSSs of more than fivefold upregulated genes (Figure 4D, G).

### MAMA successfully returned known cis-elements in A. thaliana

To investigate whether MAMA can predict *cis*-elements in other plants, we also applied it to *A. thaliana* microarrays. In microarray data generated from *A. thaliana* subjected to NaCl stress (Dinneny et al. 2008), the motif containing an abscisic acid (ABA)-responsive element (ABRE; ACGTG[G/T]C), which is a *cis*-element responsive for ABA, dehydration, low temperature, and high salinity (Narusaka et al. 2003), yielded the highest MAMA score (Figure 4B, Additional file 8 online). The motif containing a dehydration-responsive element (DRE; [A/G]CCGAC), which is involved in dehydration- and high salinity-responsive gene expression (Narusaka et al. 2003), recorded the sixth highest MAMA score (Additional file 8 online). Motifs including ZDRE, ABRE, and DRE consensus sequences were found at particularly high frequencies within 500 bp upstream of the TSSs of over twofold upregulated genes (Figure 4E, F, H, I).

### Accuracy of the MAMA method

**Figure 5:**
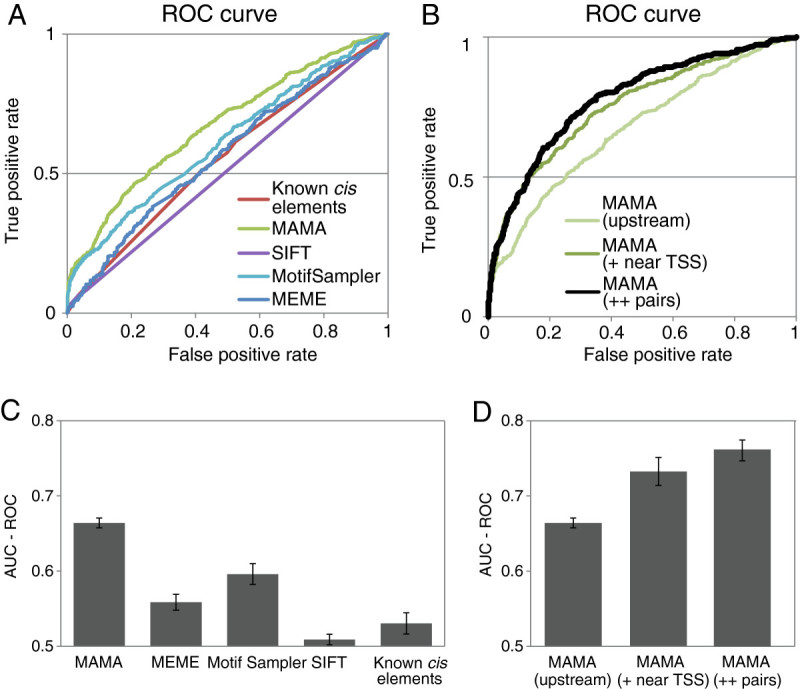
**Performance of transcription-prediction models built for predicted motifs. A**, Models generated by motifs predicted by MAMA, existing methods, or known *cis*-elements of the upregulation of transcription in rice roots under iron deficiency. The best model of each method is shown. **B**, Best predictive-transcription models using only the presence of motifs predicted by MAMA upstream of TSS (upstream), with the presence of motifs predicted from sequences -50 bp and +150 bp from the TSS (+ near TSS) and with motif pairs upstream and near the TSS (++ pairs). **C**, Comparison of putative transcription models generated by each method. Model performance was measured by the area under receiver operating characteristic curves (AUC-ROC). For each method, 10 SVM runs were conducted. **D**, Comparison of putative transcription models between only upstream sequences (upstream) and sequences -50 bp and +150 bp from the TSS (+ near TSS) and with motif pairs (++ pairs).

To evaluate how strongly these predicted motifs “explain” the regulation of transcription, we applied a machine-learning algorithm to simulate gene expression (Zou et al. 2011). This algorithm builds an expression-simulation model and classifies genes as putatively inducible and non-inducible based on the presence of *cis*-elements. Putative inducible genes were compared with genes upregulated more than twofold on a microarray to check their accuracy. Motifs predicted by MAMA, Motif Sampler, MEME, and SIFT from the top 50 genes upregulated by Fe deficiency using default settings, and the binding sequences of IDEF1, IDEF2, and OsIRO2 were used as putative and known *cis*-elements, respectively. The ratio of putative inducible to upregulated genes was assigned the “true positive rate,” and the ratio of non-upregulated to putative inducible genes was the “false positive rate” in a receiver operating characteristic (ROC) curve (Figure 5A). The area under the curve ROC (AUC-ROC) was used to check the accuracy of the model and optimization parameters (Methods).

A transcription-simulation model built on the motifs predicted by MAMA showed the best performance (Figure 5A). Additionally, the best simulation model was improved when motifs predicted from sequences 50 bp upstream and 150 bp downstream of TSSs (near TSS) by MAMA were added to the motifs predicted in sequences 500 bp upstream of TSSs (upstream; Figure 5B). Furthermore, the best simulation model improved further when motif pairs predicted from sequences upstream and near the TSS that were enriched upstream and near the TSS of regulated genes were added (Figure 5B). When randomly selected gene sets were applied to this algorithm 10 times, the AUC-ROC of MAMA was significantly higher than that of the other methods (Figure 5C). The AUC-ROC improved significantly when the model was built on motifs predicted from both the upstream sequence and sequences around TSSs (Figure 5D; + near TSS). When the presence of several motif pairs was added, the AUC-ROC tended to improve (Figure 5D; ++ pairs). When 100 motif pairs were added, the AUC-ROC was significantly impaired (Additional file 9).

After optimization, the number of genes accurately categorized was 87.9% from microarray data on *O. sativa* subjected to Fe deficiency (13,779.4 genes were accurate on an average of five tests; the number of genes used in test data was 15,676), 97.2% from microarray data on *O. sativa* subjected to Zn deficiency (14,357.2; 14,769), and 93.3% from microarray data on *A. thaliana* subjected to NaCl stress (9,691.6; 10,385).

### MAMA successfully predicted the functional cis-motifs

Motifs predicted by MAMA from microarray data of *O. sativa* subjected to Fe deficiency explained more than 87% of the transcription regulation accurately. Of the top 11 motifs extracted, four overlapped with *cis*-elements that were experimentally identified previously, such as IDEF1BS, OsIRO2BS, and IDEF2BS (Kobayashi et al. 2007; Ogo et al. 2007, 2008). The IDEF1BS motif was found at a high frequency in Fe deficiency-upregulated genes (Figure 2B). Moreover, it frequently occurred between 50 and 400 bp upstream of the TSSs of Fe deficiency-inducible genes but not in the gene population as a whole (Figure 2C). OsIRO2BS and IDEF2BS were also predicted with high MAMA scores (Table 1). These motifs were specifically overrepresented between 50 and 500 bp upstream of the TSSs of regulated genes (Additional file 6 online). These data demonstrate that MAMA successfully predicted functional *cis*-elements. Furthermore, MAMA successfully predicted ZDRE, ABRE, and DRE using *O. sativa* and *A. thaliana* microarray data (Additional file 7 and 8 online; Figure 5). These results suggest that MAMA can predict functional *cis*-elements involved in various kinds of stress responses not only in rice but also in other plants.

In addition to known *cis*-elements, MAMA predicted some novel motifs as strong candidate *cis*-elements that have not been reported before. Using the microarray data of rice under Fe-deficiency stress, FAM1 was returned with the highest MAMA score (Table 1). FAM1 was specifically overrepresented between 50 and 500 bp upstream of the TSSs of regulated genes, as is the case with other known *cis*-elements (Additional file 4 online; Table 1). Therefore, FAM1 is likely a functional *cis*-element of rice under Fe-deficiency stress. Generally, deletion of an essential *cis*-element resulted in an almost complete absence of response, whereas deletion of other parts of promoters merely lowered promoter activity (Guiltinan et al. 1990; Tong et al. 2006; Kobayashi et al. 2007). This is suggestive of the existence of important *cis*-elements, other than those reported to be essential, within promoters. Novel *cis*-elements predicted by MAMA may coordinate with known *cis*-elements to improve transcription.

### MAMA predicted cis-motifs involved in the basal transcriptional machinery

The TATA-box motif recorded the third highest MAMA score (Table 1, Figure 2D) and was the most common motif within 50 bp upstream of TSSs. This is consistent with the characteristics of the TATA box (Burley and Roeder 1996). This localization was more common in genes upregulated by Fe deficiency than in the overall gene population (Figure 2F). TATA-box motifs also frequently exist upstream of genes downregulated by Fe deficiency (Figure 4E). A genome-wide analysis in yeast revealed that stress-response genes typically possess a TATA box in their promoters, whereas housekeeping gene promoters often lack this motif (Basehoar et al. 2004). Similar accumulation of the TATA box has been observed in plants (Yamamoto et al. 2011). A TATA box is a core element of the basal transcriptional machinery that regulates genes in conjunction with other *cis*-elements (Sadhale et al. 2007). Consistent with these reports, our data demonstrated that TATA-box motifs affect the response to Fe deficiency in rice by collaborating with Fe deficiency-specific transcription factors.

Downstream core elements (DCEs) were reported in yeast and mammalians downstream of TSSs, and are known to collaborate with the TATA box (Sadhale et al. 2007). Some TATA box-binding protein (TBP)-associated factors (TAFs) bind to DCEs (Sadhale et al. 2007). Our results showed that the DCEp1 (Figure 2H) motif was commonly found immediately downstream of TSSs of Fe deficiency-inducible genes. Also, the DCEp1 motif was highly co-localized with the TATA-box motif of genes upregulated by Fe deficiency (Figure 3G, H). Thus, we suggest that a unit of the basic transcription machinery, including a TATA-box motif and DCEp1 motifs, functions in the transcriptional regulation of rice under Fe-deficiency stress.

### Co-localization of cis-motifs predicted by MAMA

Notably, the TATA-box, BRE^U^-TATA motif 1, DCEp1, and IDEF1BS motifs strongly co-localized in regions upstream of Fe deficiency-inducible genes, and the separation (in bp) between them was conserved (Figure 3). IDEF1BS motifs and BRE^U^-TATA motif 1 were frequently co-localized with a separation of 50 bp (Figure 3D), suggesting that the transcription factors binding to IDEF1BS and BRE^U^-TATA motif 1 may interact. Additionally, when the separation (in bp) of motif pairs was plotted with the frequency (i.e., Figure 3B, D, F, H), the frequency often showed several peaks, and the separation (in bp) between these peaks was commonly around 150, 300, and 450 bp (Figure 3D, H). Peaks with a separation of 150 bp have been observed in many other co-localized motifs predicted from rice microarrays under Zn deficiency and from salt-stressed *A. thaliana* microarrays (Additional file 10 online). Nucleosome core particles contain approximately 150 bp of DNA (Davey et al. 2002). Moyle-Heyrman et al. (2011) reported collaborative competition between transcription factors and the nucleosome. Therefore, these 150- and 300-bp separations of co-localized motifs may indicate either collaborative or competitive binding of transcription factors and histone. Transcription factors may bind to the interspace of DNA coiled by histone.

Motif pairs improved the AUC-ROC in transcription simulation, but the difference from that without motif pairs was not significant. The motif pairs with lower *P*-values tended to improve, and those with higher *P*-values tended to impair the AUC-ROC (Additional file 9). Of the motif pairs with lower *P*-values, some improved while others impaired the AUC-ROC. Therefore, we suggest the *Nmp* (number of motif pairs used) with the highest *AoAR* (average of AUC-ROC) as a number of highly possible candidates of motif pairs that co-regulate transcription. In addition, we suggest that *Nmp* does not impair the *AoAR* as a number of possible candidates of motif pairs that co-regulate transcription.

### Parameter optimization

In parameters power *ν* (controls the sensitivity for sequence similarity), power τ (controls the sensitivity for gene expression ratio), and number of motif pairs *N*_*mp*_, the change in *ν* was affected the most (Methods: *Comparison of the effect of parameters*), whereas τ was affected second and *N*_*mp*_ was affected last. Therefore, using this method, the parameters were adjusted in this order (Methods: *Optimization of parameters*). We also evaluated the effect of *highest_r_score*, the highest limit for the *r_score* (5, 10, 50, 100), and the threshold (1.5, 2, 3), to classify upregulated and non-upregulated genes. However, the degrees of their effects were largely different and depended on which microarray data were used. Therefore, these heuristic parameters remained unoptimized (default values; *highest_r_score* = 10*,* threshold = 2). The parameter “*highest_r_score*” may reduce noise caused by signal ratios that were too high, which was frequently observed when the gene signal was low.

## Conclusions

### A model of transcriptional regulation under Fe deficiency

**Figure 6:**
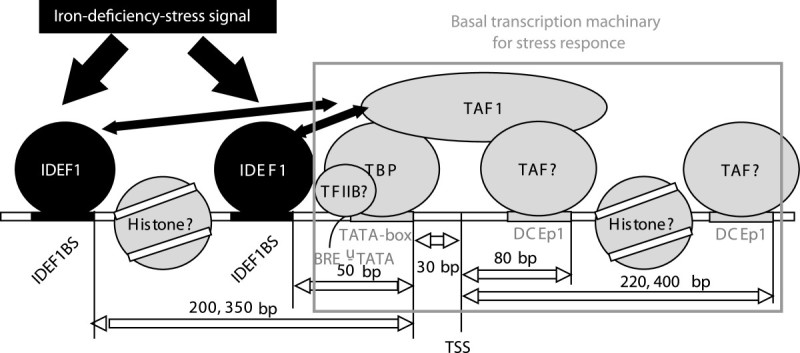
**Model of predicted motifs and factors incorporated into the regulation of gene expression in rice under iron-deficiency stress.**

Based on our predictions, we propose the following model of transcriptional regulation in rice under Fe-deficiency stress (Figure 6). The Fe-deficiency signal initially activates transcription factors involved in Fe-deficiency responses such as IDEF1. The IDEF1 binds to IDEFBS. Then, these recruit general transcription factors: TBP binds to the TATA-box motif, and TAFs to DCEp1. TFIIB may bind to BRE^U^-TATA-box motifs. TFIIB reportedly interacts with BRE^U^ via the helix-turn-helix (HTH) domain, although this domain is not conserved in yeasts and plants (Lagrange et al. 1998; Tsai and Sigler 2000). Notably, IDEF2BS and the novel motif FAM1 were co-localized with the majority of motifs in this model; however, OsIRO2BS was not (Network graph in Additional file 6), which suggested that OsIRO2 regulates genes in another model. Kobayashi et al. (2009) suggested a model that IDEF1 regulates OsIRO2 and some genes, and under the control of IDEF1, OsIRO2 regulates the other genes. Taking all these into account, we propose that OsIRO2 is regulated by IDEF1, but their targets are independent each other.

### Performance of MAMA

We compared the motifs generated by MAMA, MEME, MotifSampler and SIFT from the top 50 rice genes upregulated by Fe deficiency (Table 1, 2, Additional file 1). Motifs predicted by MAMA contributed significantly more to build transcriptional simulation model compared to those predicted using other clustering-dependent methods when motif quality was checked using the AUC-ROC of the transcription simulation model (Figure 6). Plant researchers can use MAMA to predict *cis*-motifs from microarray data on a single treatment. For example, MAMA can be applied to a microarray data under some kind of stress. MAMA optimizes parameters automatically to maximize the accuracy of simulation of gene expression. Therefore, MAMA does not require most users to determine complicated parameters. We prepared a template file for *A. thaliana* microarray ATH1. Users can run MAMA after pasting the signal ratio from the microarray data to the template file. All the calculations of MAMA were performed using Desktop PC (Dell Vostro 470 with Quadro 2000, 8GB RAM, Windows 7) and the calculation of a data set took from 11 to 54 hours. We developed the main software using GPGPU (CUDA; supported by NVIDIA GeForce (8 or higher), Tesla or Quadro series). Using the CUDA environment, optimization can be completed within 3 days. However if you do not have CUDA environment, some parameters optimization using CPU (core i7 3770) in MAMA requires several weeks.

### Future development

We expect MAMA to increase our understanding of the complex regulation of gene expression in higher eukaryotes from the co-localizations and the separation (in bp) between them. A method developed by Huttenhower et al. (2009) generates regulatory modules: co-regulated genes, the conditions under which they are co-regulated and sequence-level regulatory motifs. Using COALESCE, the genes upregulated under iron deficiency may be separated into a subcluster regulated by a model including IDEF1BS and another cluster regulated by another model including OsIRO2BS, and we may analyze more specifically about the regulation occurred in each subgroup. It is necessary to prepare microarray data similar to the one under iron deficiency to perform COALESCE effectively. MAMA and all the programs used in this study are available for download at http://park.itc.u-tokyo.ac.jp/pbt/MAMA.

## Methods

### Definitions

*N* is the number of genes from the microarray data, *N*(A) is the number of genes containing motif A, *N*(!A) is the number of genes that do not contain motif A, *N*(UP) is the number of genes upregulated more than twofold, *CR*(A) is the cover ratio of motif A, and *CR*(A) = *N*(A)/*N.*

### Preparation of sequences and microarray data

The rice genome sequence (IRGSP1.0) was downloaded from the RAP-DB Web site (http://rapdb.dna.affrc.go.jp/). Genes possessing identical promoters were treated as a single gene (ID). In these cases, the geometric mean of their gene expression ratios was used. Ratios of expression in Fe-deficient and -sufficient plants, obtained using microarrays (Ogo et al. 2008) (cv. Tsukinohikari), were used in subsequent studies. Microarray data on rice root under Zn-deficient and -sufficient plants were obtained from a published paper (Suzuki et al. 2012) (cv. Nipponbare). The genome sequence of *A. thaliana* and gene annotation data (TAIR10) were retrieved from TAIR (http://www.arabidopsis.org). Microarray data generated using *A. thaliana* subjected to NaCl stress were obtained from a previous report (Dinneny et al. 2008). Random sequences were generated using a random sequence generator with probabilities of A:C:G:T as 0.25:0.25:0.25:0.25 (http://tandem.bu.edu/rsg.html).

### Calculation of MAMA scores

MAMA was developed to identify motifs that were frequently present in upstream regions of regulated genes. This method initially lists every 8-bp sequence upstream of regulated genes as candidate sequences. Candidate sequences were extracted from the 50 most highly upregulated genes from the microarray analyses. First, MAMA assigned each gene a number (*n*). MAMA assigned each candidate sequence a *MAMA score*, which was designed to reflect the enrichment of the frequency and similarity of the candidate sequence in highly upregulated genes in microarray analyses (Additional file 2). The lengths (in bp) of sequences showing identity to part or all of the candidate sequence, as well as the separation (in bp) between the two identical sequences, were used in the calculations. The length of the *x*^th^ identical part was defined as *hx*. The separation (in bp) between two identical sequences (*x*^th^ and *y*^th^) was defined as *d*_*x,y*_. The *MAMA score* for each candidate motif was calculated using the following formula:

1 $$\mathrm{MAMA}\;\mathrm{score}=\frac{\sum_{n=1}^{N}\left(h\_\mathrm{score}\left(n\right)\times r\_\mathrm{score}\left(n\right)\right)}{\sum_{n=1}^{N}h\_\mathrm{score}\left(n\right)}.$$

The *h_score* is calculated according to the following procedure:

2 $$\begin{array}{l} h\_\mathrm{score}=\{\left(h1\right)!+\left(h2\right)!+\ldots +\left(\mathrm{hx}\right)!\\ \;+h1*h2/\left(â*d_{1,2}+1\right)\\ \;+h1*h3/\left(â*d_{1,2}+1\right)/\left(â*d_{2,3}+1\right)+\ldots \\ \;+h1*\mathrm{hx}/\left(â*d_{1,2}+1\right)/\ldots /\left(â*d_{\left(x-1\right),x}+1\right)\\ \;+\;h2*h3/\left(â*d_{2,3}+1\right)\\ \;+\left(h2\right)*h4/\left(â*d_{2,3}+1\right)/\left(â*d_{3,4}+1\right)+\ldots +\\ \;+h2*\mathrm{hx}/\left(â*d_{2,3}+1\right)/\ldots /\left(â*d_{\left(x–1\right),x}+1\right)+\ldots \\ \;+\;h\left(x–1\right)*\mathrm{hx}/{\left(â*d_{\left(x–1\right),x}+1\right)\}}^{v} \end{array}$$

The *h_score* was designed to calculate the similarity of a promoter to a candidate sequence. In the present study, both DNA strands ware used to calculate *h_scores*. Every part of a promoter with the same length as a candidate sequence was compared with candidate sequences, and the highest *h_score* in a promoter was selected as the *h_score*(*n*) of a gene(*n*). Uninterrupted identity to the candidate sequence and short separations between identical sequences yielded higher *h_scores*. To control the effect of the separation between them, penalty *â* was set. To control the sensitivity for sequence similarity on the MAMA score, the result is raised to the power *v*. For each gene, the *r_score*(*n*) represents the microarray gene expression ratio. To control the influence of expression ratio on the MAMA score, a threshold *highest_r_score* was set. In cases in which the gene expression ratio exceeded this threshold, the *r_score* was set to the threshold. The threshold *highest_r_score* was set to 10.0 (default). When calculating correlations between sequence and upregulation, MAMA offers the option of removing downregulated genes from the analysis or setting the *r_score* to 1.0 or 1/expression ratio. *r_scores* for downregulated genes were set to 1.0 (default). To control the sensitivity for gene expression ratios on the MAMA score, the *r_scores* were raised to the power τ.

### Grouping of similar sequences

High-scoring candidate sequences were identified after MAMA score calculation. For the 5% highest-scoring candidate sequences, similar and lower-scoring candidate sequences were grouped into the same motif group as the higher-scoring one. In the present study, two mismatched bases were permitted (i.e., ≥6 bp identity to the higher-scoring candidate motif).

### Evaluation of predicted motifs using a transcription-prediction algorithm

To evaluate the correlation of the presence of predicted motifs with upregulation of genes, we used a classification algorithm by Support Vector Machine (SVM; Joachims 1999). All SVM runs were performed by LIBSVM3.1 (Fan et al. 2005). The problem “how predicted motifs may be used to simulate upregulation of transcription” was formalized as a machine-learning classification problem (Zou et al. 2011). We were interested in assigning genes into two classes, namely, inducible (1) and non-inducible (-1) based on a feature vector describing the presence (1) and absence (0) of motifs and motif pairs in a gene. For training of the models, genes upregulated more than twofold by treatment were used as positive examples. Genes that were not upregulated more than twofold were used as negative examples. For each SVM run, genes were randomly separated into training and test sets. Because the number of positive examples was much smaller than that of negative examples, random undersampling of negative examples was applied to improve the performance of the highly imbalanced data (Tang et al. 2009). *Ru* (proportion of negative samples) was set to 1/16 of negative samples. For each training set, the optimal parameters for *C* (trade-off between training error and margin) and γ (gamma in the kernel function) were examined by grid search. The performance of the classifier was measured by the AUC-ROC during the optimization, and optimal parameters that resulted in the highest AUC-ROC were applied to test sets.

### Evaluation of motif co-localization

The *P*-values for the co-localization of motifs were calculated using Pearson’s chi-square test using the following formula:

3 $$X^{2}=\sum \frac{{\left(\mathrm{Oi}-\mathrm{Ei}\right)}^{2}}{\mathrm{Ei}},$$

A region from 500 bp upstream to 150 bp downstream of TSSs was used to evaluate the co-localization of motif pairs (e.g., motif A and B). In the above equation, *Oi* represents the observed number of *N*(A|B|UP), *N*(!A|B|UP), *N*(A|!B|UP), *N*(!A|!B|UP)…while *Ei* represents the expected number, *N*(UP)*N*(A|B|!UP)/N(!UP), *N*(UP)*N*(!A|B|!UP)/N(!UP), *N*(UP)*N*(A|!B|!UP)/N(!UP), *N*(UP)*N*(!A|!B|!UP)/N(!UP); two enrichments of motif A were simultaneously evaluated as *EN1* and *EN2*. *EN1* was defined as *N*(A|B|UP)/*N*(B|UP) divided by *N*(A|!B|UP)/*N*(!B|UP). *EN2* was defined as *N*(A|B|UP)/*N*(B|UP) divided by *N*(A|B|!UP)/*N*(B|!UP). Enriched motif pairs were defined as motif pairs of which *EN1* and *EN2* were greater than 1. When the number of motif pairs used in MAMA was set to *N*_*mp*_, motif pairs with the top *N*_*mp*_ lowest *P*-value were used for the simulation of gene expression. If motif A and motif B contained identical sequences, co-localization was not evaluated.

### Optimization of MAMA parameters

Parameters power *ν* (controls the sensitivity for sequence similarity), power τ (controls the sensitivity for the gene expression ratio), and number of motif pairs *N*_*mp*_ applied for the SVM were optimized one by one in this order. These parameters started from 1, 1, and 0, respectively, and increased by 1 after a set of simulations. During the optimization of power *ν*, power τ *=* 1, 2, 3, 4, and 5 were tested five times each, and the average AUC-ROC (= *AoAR*_(v)_) was calculated from these 25 simulations. After simulation with increased power *ν*, if *AoAR*_(v)_ < *AoAR*_(v–1)_, then the optimized power *ν* was set to*ν –* 1; otherwise, power *ν* was increased further. An increase in power τ reached a plateau of the AUC-ROC value. During the optimization of power τ, power τ *=* power τ, power τ *+* 1, power τ *+* 2, power τ *+* 3, and power τ *+* 4 was tested five times each, and the slope of the AUC-ROC (*SoAR*_(τ)_) was calculated using power τ and the AUC-ROC from these 25 simulations. If *SoAR*_(τ)_ was not defined or bigger than the defined maximum value of *SoAR*_(τ)_ (*MaxSoAR*_(τ)_), then *MaxSoAR*_(τ)_ was set to *SoAR*_(τ)_. After the increase in power τ, if *SoAR*_(τ)_ < (*MaxSoAR*_(τ)_/2), then the optimized power τ was set to τ; otherwise, power *ν* was increased further. If τ was more than five, the integral 5/τ was added to τ*.* During the optimization of *N*_*mp*_, 1, 2, 3, 5, 10, 20, 30, 50, 100, and 200 were tested five times each. The average AUC-ROC *AoAR*(_*Nmp*_) was calculated for each *N*_*mp*_ value (10 tests each), and the *N*_*mp*_ with the highest *AoAR*(_*Nmp*_) was set to optimized *N*_*mp*_.

### Comparison of the effect of parameters to AUC-ROC

Initially, we tested parameters *ν* (1, 2, 3, 4, 5), τ (1, 2, 3, 4, 5, 6, 7, 8, 9, 10, 11, 12, 13, 14, 15), and *Nmp* (1, 2, 3, 5, 10, 20, 30, 50, 100, 200) using microarray data from rice subjected to Fe deficiency and Zn deficiency, and *A. thaliana* subjected to NaCl stress. For each *ν*, the average AUC-ROC calculated using all the tested power τ and *Nmp* was compared (*AoAR*(*ν*, ∑τ, ∑*Nmp*)). The difference between the highest *AoAR*(*ν*, ∑τ, ∑*Nmp*) and lowest *AoAR*(*ν*, ∑τ, ∑*Nmp*) was evaluated as the effect of *ν*. Similarly, the difference between the highest *AoAR*(∑*ν*, τ, ∑*Nmp*) and lowest *AoAR*(∑*ν*, τ, ∑*Nmp*) was evaluated as the effect of τ. The difference between the highest *AoAR*(∑*ν*, ∑τ, *Nmp*) and lowest *AoAR*(∑*ν*, ∑τ, *Nmp*) was evaluated as the effect of *Nmp*.

### Prediction of cis-elements with existing methods

MEME (Bailey and Elkan 1994), Motif Sampler (Thijs et al. 2001), and SIFT (Hudson and Quail 2003) were used to compare the result of predicted *cis*-elements. Also, 500-bp upstream sequences from the TSS of the top 50 the most upregulated genes in microarray data on rice subjected to Fe deficiency were used as input. Background data were generated from 500-bp upstream sequences from the TSSs of rice genes, of which the gene expression ratio was between 0.8 and 1.2. Most parameters remained as default values. If word size was required, the word size was set to 8. The number of outputs was set to 1,250, and 1,250 motifs each were used to simulate gene expression by SVM.

## Electronic supplementary material

Additional file 1: Motifs predicted by MotifSampler, MEME and SIFT. (XLSX 33 KB)

Additional file 2: About MAMA score, how to calculate MAMA score and examples. (PDF 398 KB)

Additional file 3: Enrichment and Annotation of predicted by MAMA using microarray data from iron-deficient. (XLSX 30 KB)

Additional file 4:**Characterization of motifs predicted using microarray data from rice roots subjected to Fe deficiency.**(PDF 491 KB)

Additional file 5: Enrichment and Annotation of predicted from a region 50 bp upstream to 150 bp downstream of TSS. (XLSX 19 KB)

Additional file 6: Enrichment of motif pairs. (XLSX 271 KB)

Additional file 7: Motifs predicted using microarray data from rice roots subjected to Zn deficiency. (XLSX 82 KB)

Additional file 8: Motifs predicted using microarray data of *A. thaliana* subjected to NaCl stress. (XLSX 80 KB)

Additional file 9: Number of motif pairs used (*Nmp*) and the value of AUC-ROC. (PDF 305 KB)

Additional file 10: Separations (in bp) between motifs predicted from microarray data of *O. sativa* subjected to zinc deficiency and *A. thaliana* subjected to NaCl stress. (PDF 452 KB)

Below are the links to the authors’ original submitted files for images.

Authors’ original file for figure 1

Authors’ original file for figure 2

Authors’ original file for figure 3

Authors’ original file for figure 4

Authors’ original file for figure 5

Authors’ original file for figure 6

## Abbreviations

TSS: Transcription start site
MAMA: Microarray-associated motif analyzer
TF: Transcription factor
TFBS: Transcription factor binding site
IDE1 and 2: Iron-deficiency responsive element 1 and 2
IDEF1 and 2: IDE1-binding factor and IDE2-binding factor
IDEF1BS IDEF2BS and OsIRO2BS: Motif containing binding sequence of IDEF1, IDEF2 and OsIRO2
FAM: Fe deficiency-associated motif 1
DCEp1: Putative downstream core element 1
MEME: Multiple Em for Motif Elicitation
ROC curve: A receiver operating characteristic curve
AUC-ROC: The area under the curve of ROC curve
SVM: Support vector machine.
