# Supplementary material for: Development of a novel prediction method of cis-elements to hypothesize collaborative functions of cis-element pairs in iron-deficient rice
Source: Rice (N Y). 2013 Sep 22;6:22. doi: 10.1186/1939-8433-6-22 (PMC4883709; doi:10.1186/1939-8433-6-22)
Supplement: Supplementary file 2 — Additional file 2: About MAMA score, how to calculate MAMA score and examples. (PDF 398 KB) [file 12284_2013_62_MOESM2_ESM.pdf]

About MAMA score

The MAMA score was calculated from gene expression and sequence similarities. The following examples will help clarify how this score is related to gene expression and sequence similarities.

How to calculate MAMA score

$CR = N(x|y)/N(y)$

CR: Cover ratio
N(x|y): number of genes that have x bp of identical sequence to a candidate sequence and upregulated y-folds
N(y): number of genes upregulated y-folds

Cover ratio of sequence

10%	30%	60%	90%

Sequence similarity

Maximum number of base pairs identical to a part of promoter (x)

Gene expression — Folds up-regulated (y)

y \ x	8	7	6	5
3	90%	90%	90%	90%
2	30%	60%	60%	90%
1	10%	30%	60%	90%

*MAMA score $= \sum(CR \cdot x \cdot y) / \sum(CR \cdot x)$

* In the paper, x was replaced with h_score, y was replaced with r_score, and each gene (1/1) was used instead of the cover ratio.

$$\{(0.9\cdot8\cdot3)+(0.9\cdot7\cdot3)+(0.9\cdot6\cdot3)+(0.9\cdot5\cdot3)$$
$$+(0.3\cdot8\cdot2)+(0.6\cdot7\cdot2)+(0.6\cdot6\cdot2)+(0.9\cdot5\cdot2)$$
$$+(0.1\cdot8\cdot1)+(0.3\cdot7\cdot1)+(0.6\cdot6\cdot1)+(0.9\cdot5\cdot1)\}$$
$$/\{(0.9\cdot8)+(0.9\cdot7)+(0.9\cdot6)+(0.9\cdot5)$$
$$+(0.3\cdot8)+(0.6\cdot7)+(0.6\cdot6)+(0.9\cdot5)$$
$$+(0.1\cdot8)+(0.3\cdot7)+(0.6\cdot6)+(0.9\cdot5)\} = 2.25$$

MAMA score and examples

Candidate sequence					Score	Exsample
Candidate sequence A (×)	90%	90%	90%	90%	2.0	Common sequence
	90%	90%	90%	90%		
	90%	90%	90%	90%		
Candidate sequence B (×)	10%	30%	60%	90%	2.0	Completely at random<br>Sequences 8 bp identical are rare<br>Sequences 5 bp identical are common
	10%	30%	60%	90%		
	10%	30%	60%	90%		
Candidate sequence C (△)	30%	30%	60%	90%	2.05	8 bp identical sequence<br>is enriched in genes upregulated threefold<br>when it is compared from that of onefold
	10%	30%	60%	90%		
	10%	30%	60%	90%		
Candidate sequence D (○)	30%	60%	60%	60%	2.25	8 bp identical sequence<br>is enriched in genes upregulated threefold<br>7 bp identical sequence<br>is enriched in genes upregulated two- and threefold<br>6 bp identical sequence<br>is enriched in genes upregulated threefold
	10%	30%	30%	60%		
	10%	10%	30%	60%		

MAMA scores become higher if identical sequences are enriched in upregulated genes.

Kakei et al. Additional file 2
