# Supplementary material for: Development of a novel prediction method of cis-elements to hypothesize collaborative functions of cis-element pairs in iron-deficient rice
Source: Rice (N Y). 2013 Sep 22;6:22. doi: 10.1186/1939-8433-6-22 (PMC4883709; doi:10.1186/1939-8433-6-22)
Supplement: Supplementary file 4 — Additional file 4:Characterization of motifs predicted using microarray data from rice roots subjected to Fe deficiency.(PDF 491 KB) [file 12284_2013_62_MOESM4_ESM.pdf]

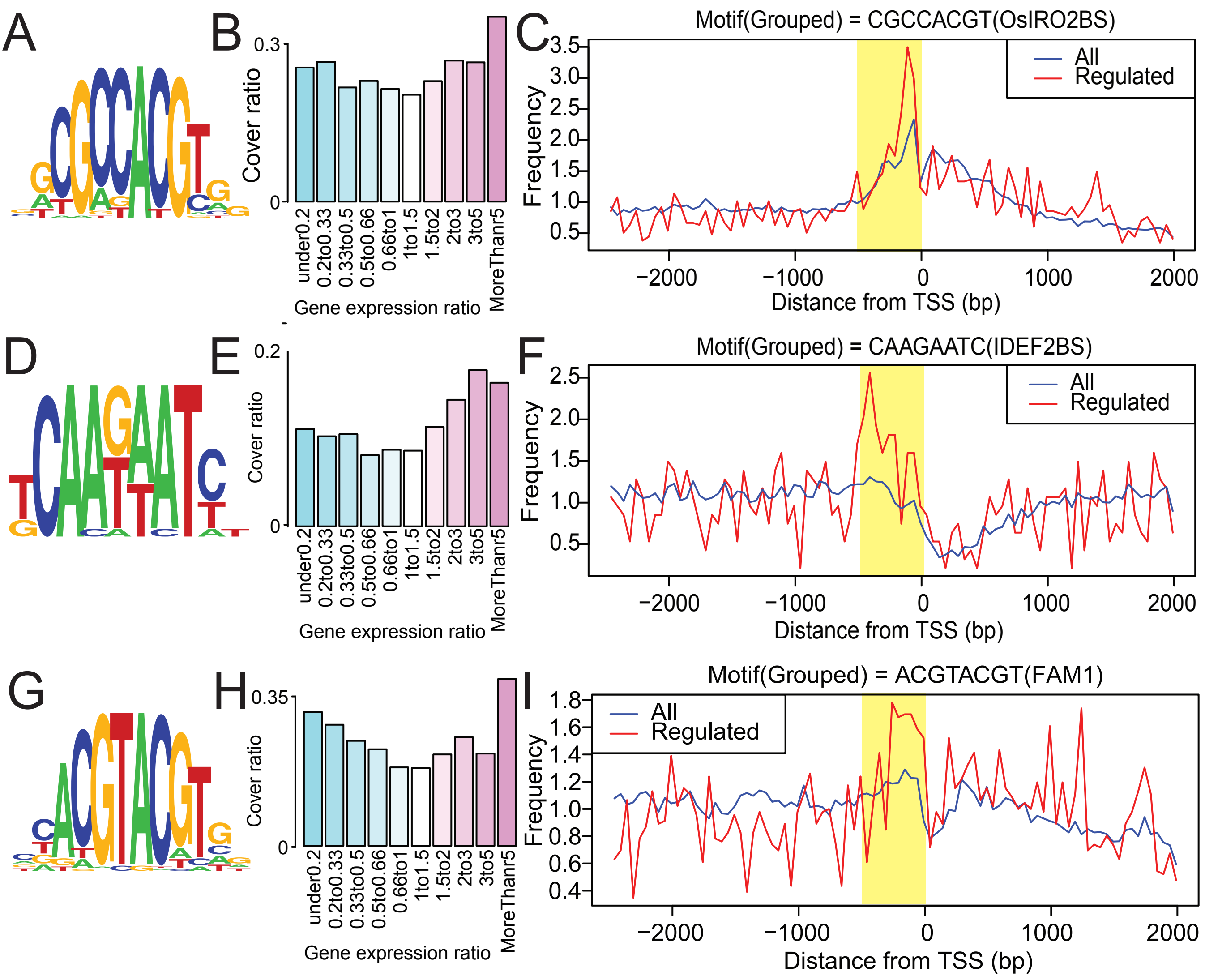

Kakei et al. Additional file 4

Additional file 4. Characterization of motifs predicted using data from rice roots subjected to iron deficiency. A, Logo represents aligned sequences included in the CGCCACGT motif (OsIRO2BS motif). Heights of A/C/G/T in the logo represent the frequency of bases at that position. B, Cover ratio (CR) of the OsIRO2BS motif in the 500-bp upstream regions. C, Distribution of the OsIRO2BS motif. Numbers of OsIRO2BS motifs were counted at a region from 3,000 bp upstream to 2,000 bp downstream of the TSS. The blue line represents the frequency of OsIRO2BS in all genes. The number of OsIRO2BS motifs in a 50-bp window in genes upregulated by iron deficiency (red) or all genes (blue) divided by the number of upregulated (*N*(UP) = 895) or all genes (*N* = 31,348); next, the frequency was normalized by the average frequency of all genes from 3,000 bp upstream to 2,000 bp downstream of the TSS. D, Logo of the CAAGAATC motif (IDEF2BS motif). E, Cover ratio of the IDEF2BS motif. F, Distribution of the IDEF2BS motif. G, Logo of the ACGTACGT motif (FAM1 motif). H, Cover ratio of the FAM1 motif in the 500-bp upstream regions. I, Distribution of the FAM1 motif.
