# Supplementary material for: Development of a novel prediction method of cis-elements to hypothesize collaborative functions of cis-element pairs in iron-deficient rice
Source: Rice (N Y). 2013 Sep 22;6:22. doi: 10.1186/1939-8433-6-22 (PMC4883709; doi:10.1186/1939-8433-6-22)
Supplement: Supplementary file 9 — Additional file 9: Number of motif pairs used (Nmp) and the value of AUC-ROC. (PDF 305 KB) [file 12284_2013_62_MOESM9_ESM.pdf]

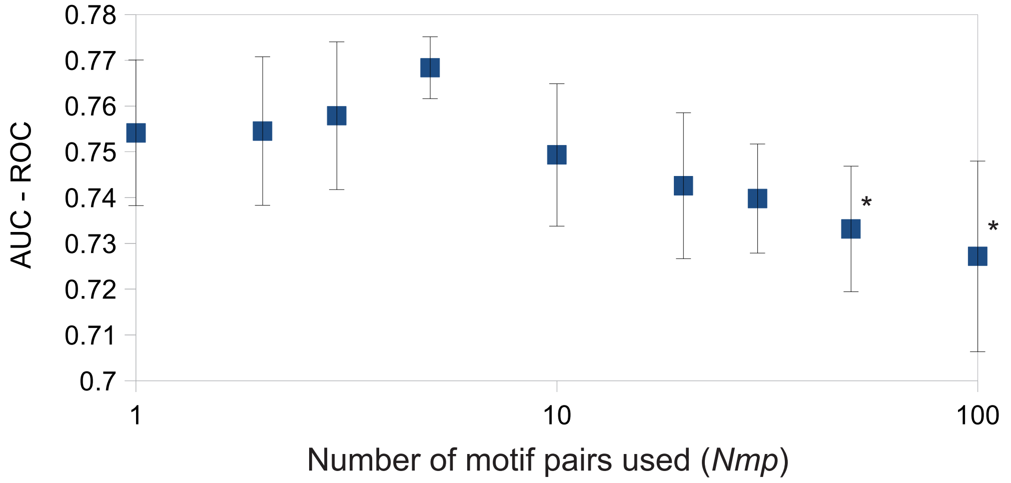

Kakei et al. Additional file 7

Additional file 7. Number of motif pairs used (*Nmp*) and the value of AUC-ROC. * *P* value < 0.05 by student-t test. AUC-ROC of ten runs with motif pairs were compared from that without motif pairs. n=10. mean ±S.D.
