# Supplementary material for: Development of a novel prediction method of cis-elements to hypothesize collaborative functions of cis-element pairs in iron-deficient rice
Source: Rice (N Y). 2013 Sep 22;6:22. doi: 10.1186/1939-8433-6-22 (PMC4883709; doi:10.1186/1939-8433-6-22)
Supplement: Supplementary file 10 — Additional file 10: Separations (in bp) between motifs predicted from microarray data of O. sativa subjected to zinc deficiency and A. thaliana subjected to NaCl stress. (PDF 452 KB) [file 12284_2013_62_MOESM10_ESM.pdf]

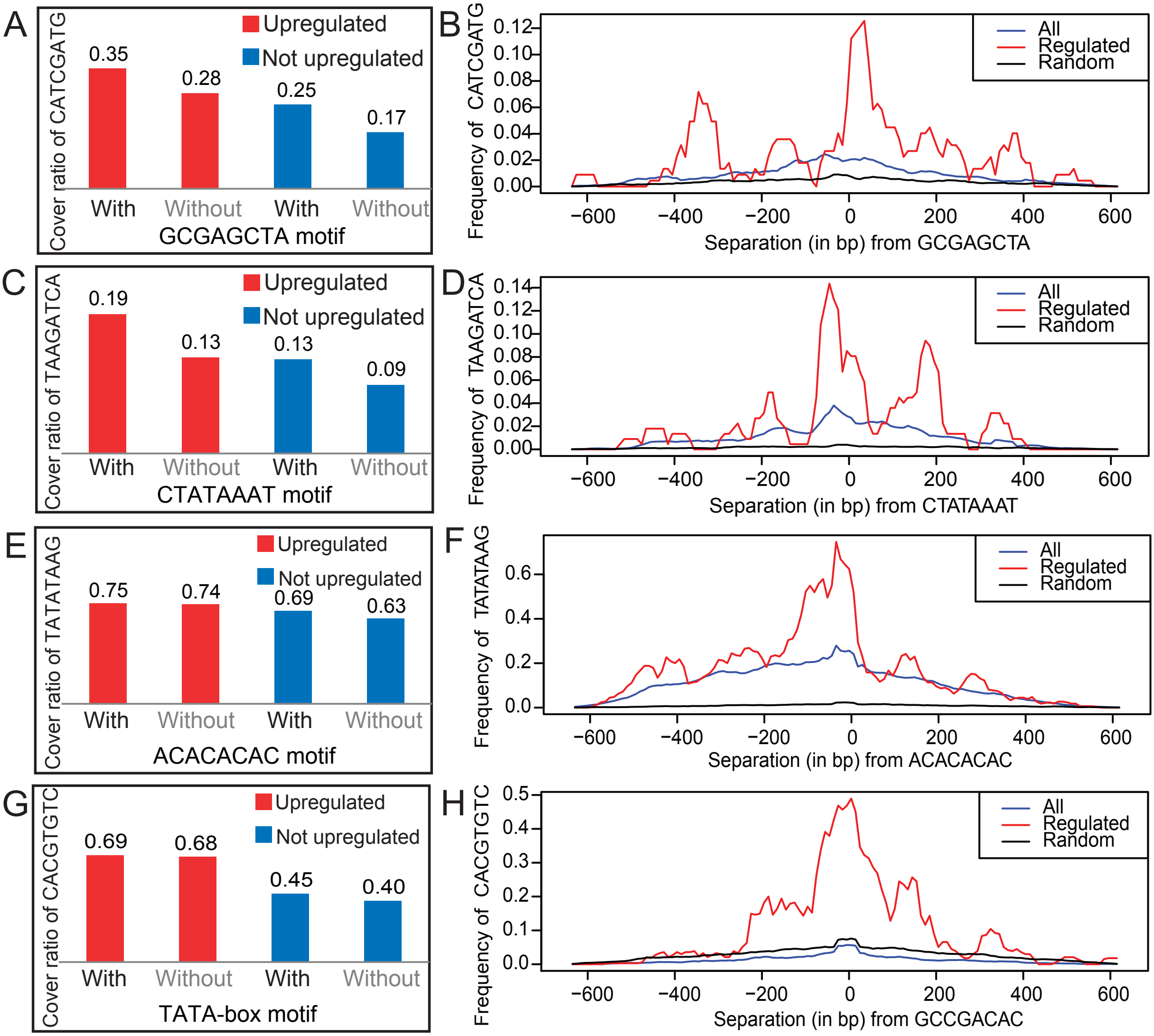

Kakei et al. Additional file 8

Additional file 8. Separations (in bp) between motifs predicted from microarray data of *O. sativa* subjected to zinc deficiency (A, B, C, D) and *A. thaliana* subjected to NaCl stress (E, F, G, H). A, Cover ratio of the CATCGATG motif with (*CR*(CATCGATG|GCGAGCTA|UP), *CR*(CATCGATG|GCGAGCTA|!UP)) and without the GCGAGCTA motif (*CR*(CATCGATG|!GCGAGCTA|UP), *CR*(CATCGATG|!GCGAGCTA|!UP)). B, Separations between the CATCGATG and GCGAGCTA motifs. Separations between the CATCGATG and GCGAGCTA motifs were calculated, and the number of CATCGATG motifs co-localizing with GCGAGCTA motifs in a region 500 bp upstream and 150 bp downstream of the TSSs was counted. Black lines, frequency of motifs in random and 650-bp sequences; blue lines, number of motifs in all genes; red lines, number of motifs in genes upregulated over twofold in response to zinc deficiency. Frequency represents the number of CATCGATG motifs in each ±25-bp window in random sequences, upregulated genes, and all genes divided by the number of random sequences (29758), upregulated genes (223), and all genes (29758). C, Cover ratio of the TAAGATCA motif with the CTATA-AAT motif 1. D, Separations between the TAAGATCA motif and CTATAAAT motif 1. E, Cover ratio of the TATATAAG motif with the ACACACAC motif 1. F, Separations between the TATATAAG motif and ACACACAC motif 1. G, Cover ratio of the CACGTGTC motif with the GCCGACAC motif. H, Separations between the CAC-GTGTC motif and GCCGACAC motif.
