# Supplementary figures and images for: Development of a novel prediction method of cis-elements to hypothesize collaborative functions of cis-element pairs in iron-deficient rice
Source: Rice (N Y). 2013 Sep 22;6:22. doi: 10.1186/1939-8433-6-22 (PMC4883709; doi:10.1186/1939-8433-6-22)

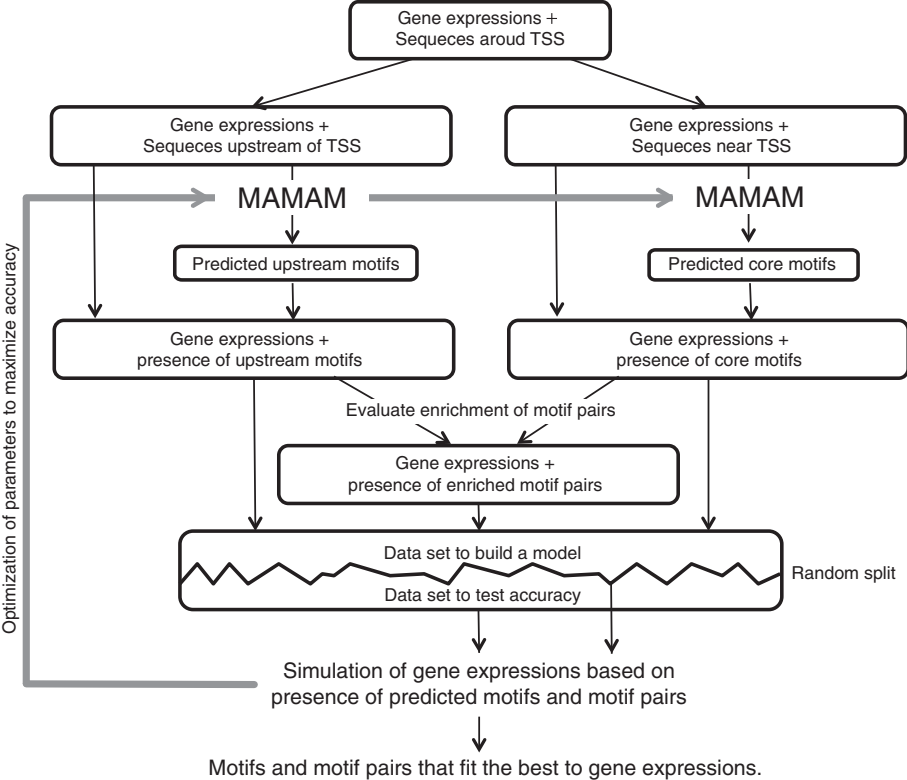

Supplement: Supplementary file 11 — Authors’ original file for figure 1 [file 12284_2013_62_MOESM11_ESM.pdf]

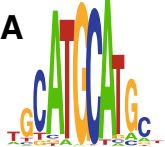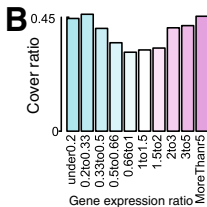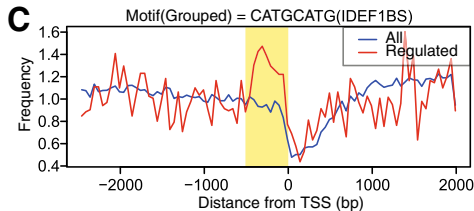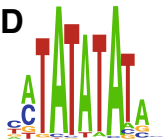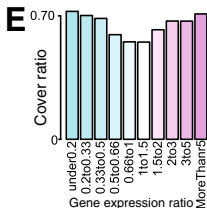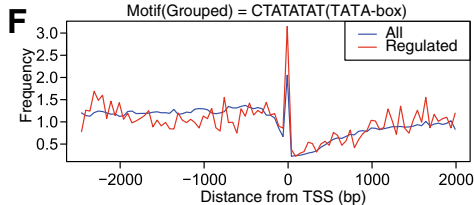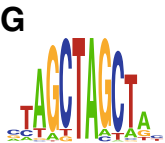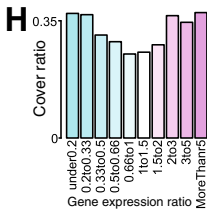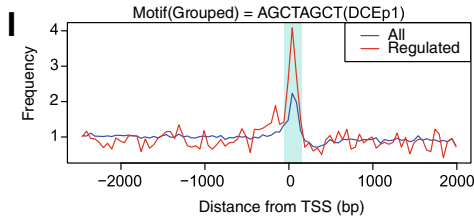

Supplement: Supplementary file 12 — Authors’ original file for figure 2 [file 12284_2013_62_MOESM12_ESM.pdf]

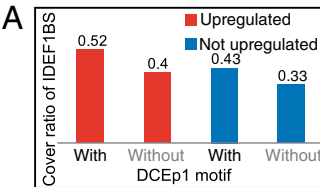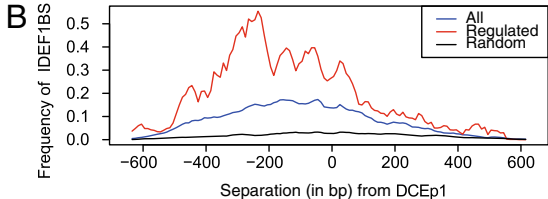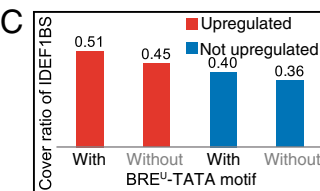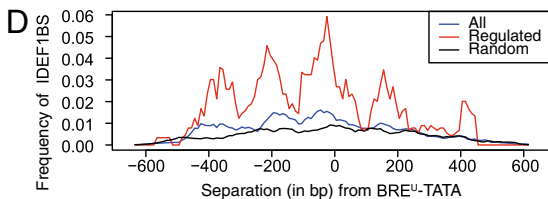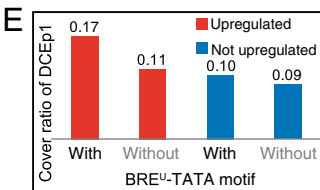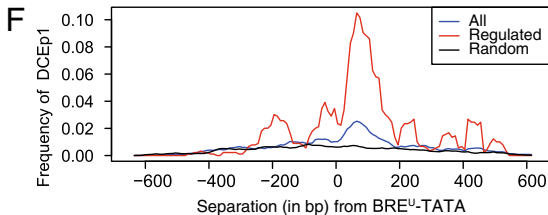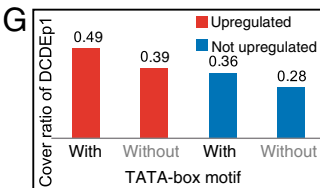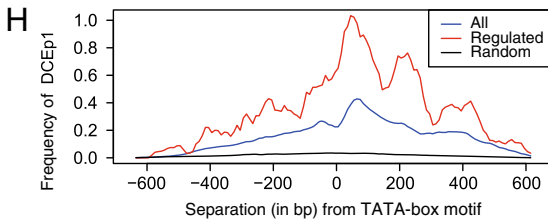

Supplement: Supplementary file 13 — Authors’ original file for figure 3 [file 12284_2013_62_MOESM13_ESM.pdf]

**A**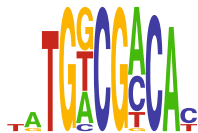**D**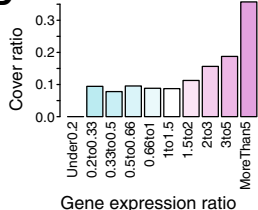**G**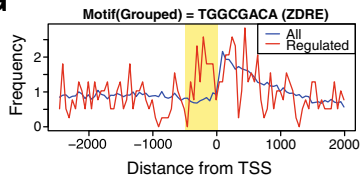**B**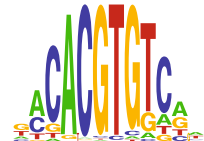**E**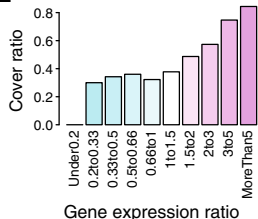**H**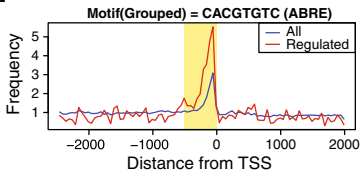**C**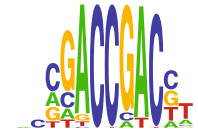**F**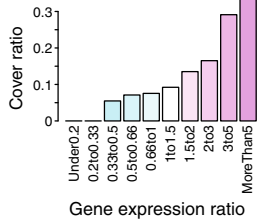**I**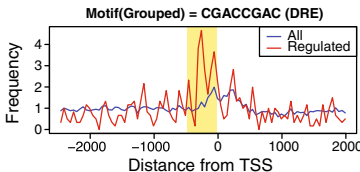

Supplement: Supplementary file 14 — Authors’ original file for figure 4 [file 12284_2013_62_MOESM14_ESM.pdf]

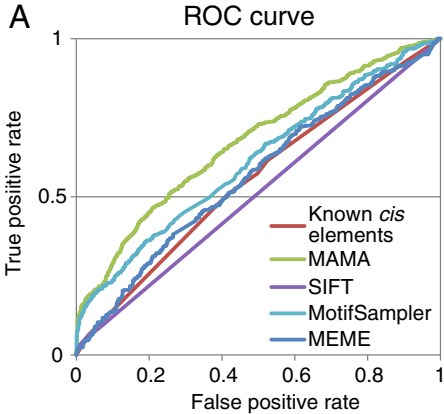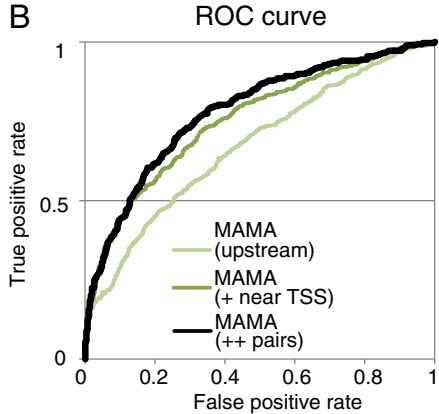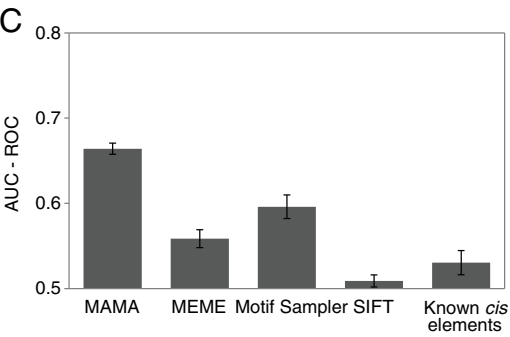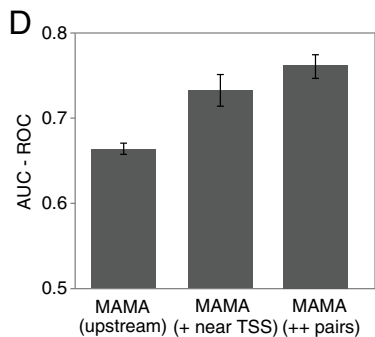

Supplement: Supplementary file 15 — Authors’ original file for figure 5 [file 12284_2013_62_MOESM15_ESM.pdf]

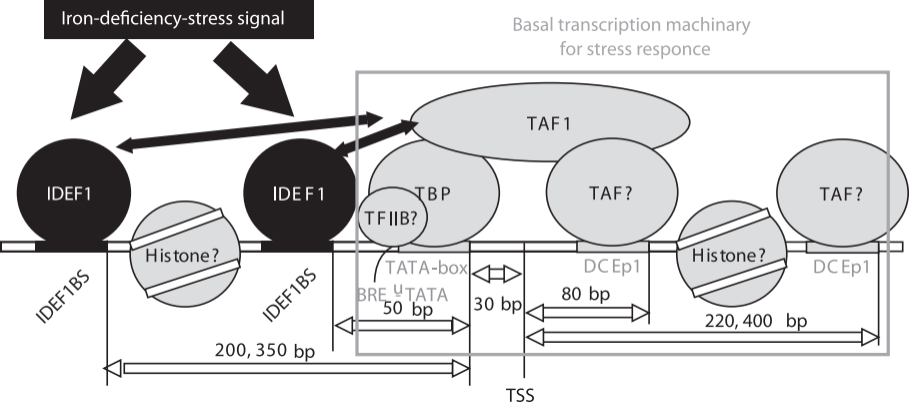

Supplement: Supplementary file 16 — Authors’ original file for figure 6 [file 12284_2013_62_MOESM16_ESM.pdf]
